# STING Modulating ER‐Phagy in the Prelimbic Cortex Neurons Contributed to Neuropathic Pain and Emotional Comorbidity

**DOI:** 10.1002/cns.70995

**Published:** 2026-06-22

**Authors:** Yongda Liu, Xu Yang, Shihui Kuai, Xi Chen, Zhibin Wang, Zhongsheng Yang, Xiaochuan Guo, Xin He, Ping Zhao

**Affiliations:** ^1^ Department of Anesthesiology Shengjing Hospital of China Medical University Shenyang China; ^2^ Department of Pain Management Shengjing Hospital of China Medical University Shenyang China; ^3^ Department of Neurology Shengjing Hospital of China Medical University Shenyang China

**Keywords:** antidepressants, cGAS/STING pathway, ER‐phagy, neuropathic pain, prelimbic cortex

## Abstract

**Background:**

Our previous data suggested that autophagy is crucial for neuropathic pain. The different cell types and varying degrees of regulation of STING may lead to a paradoxical effect on pain and emotions in the neuropathic pain model. Up to now, whether STING modulates neuropathic pain in PrL neurons via ER‐phagy is still unknown.

**Method:**

In this study, we investigated the effect of ER‐phagy in the prefrontal cortex (PrL) on neuropathic pain. We administered 4‐phenylbutyric acid, tunicamycin, 3‐methyladenine, and rapamycin to evaluate the interaction between endoplasmic reticulum (ER) stress and autophagy in the PrL of SNL (spinal nerve ligation). We injected AAV to investigate whether ER‐phagy modulated pain and emotional behaviors. We further explored whether STING and its pathway as a modulation target for ER‐phagy to participate in the pain process. We injected 2′3‐cGAMP and RU521 to modulate the cGAS/STING pathway in ER‐phagy in SNL mice. Moreover, we modulated STING expression and regulated the levels of ER‐phagy and the interaction between STING and LC3 in neurons through PrL AAV injections.

**Results:**

The data indicated that ER‐phagy alleviated the excessive ER stress induced by SNL in PrL through the cGAS/STING pathway. Regulating ER‐phagy in PrL neurons through AAV tools altered pain and emotion‐related behaviors. In addition, regulating STING in PrL neurons altered the comorbidity of pain and emotion. Importantly, the binding interaction between STING and LC3 in PrL neurons provides a novel target for PrL ER‐phagy.

**Conclusion:**

Enhanced ER‐phagy of PrL neurons provides analgesic, anti‐anxiety, and antidepressant effects through modulating STING in SNL mice.

## Graphical abstract

SNL induced ER‐phagy impairment and ER stress increase in the PrL neuron via STING and the STING pathway. Our data indicated that enhancing ER‐phagy in PrL neurons might provide analgesic and antidepressant effects through STING and its pathways in SNL mice.

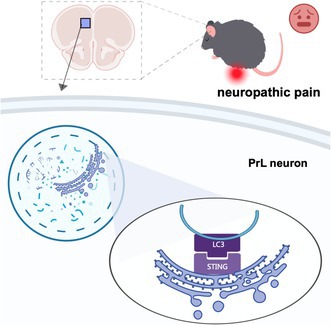

## 1 Introduction

Neuropathic pain (NP) is a complex disease accompanied by negative emotional disorders such as anxiety and depression, which increase the difficulty of treatment and highlight unmet medical needs [1]. Research has shown that mechanisms within the forelimb cortex (PrL) of the ascending and descending nociceptive system play a crucial role in neuropathic pain and comorbid depression [2, 3]. In addition, targeting PrL has the potential to become a drug and neuromodulation therapy target for neuropathic pain.

Our previous studies have shown that spinal autophagy is impaired in the NP model, and regulating autophagy may be a feasible therapeutic strategy [4]. Our proteomic sequencing data indicate that autophagy is altered in the PrL of SNL (spinal nerve ligation) mice, and according to gene ontology (GO) functional analysis, ER (endoplasmic reticulum) activity is associated with this. ER‐phagy is a selective autophagy pathway that targets damaged endoplasmic reticulum to lysosomes for degradation. By eliminating misfolded ER, it maintains secretory homeostasis and protects neurons from proteotoxic stress. ER‐phagy/reticular‐phagy serves as a rescue mechanism by eliminating damaged organelles or aging proteins after endoplasmic reticulum stress in SNL mice. Further exploration is needed to determine whether autophagy can alleviate ER stress in the PrL of SNL mice through endoplasmic reticulum autophagy. Therefore, we focused on studying the ER‐phagy and related molecular mechanisms within the PrL of neuropathic pain.

STING (Interferon Gene Stimulating Factor) is a transmembrane protein located on the endoplasmic reticulum and serves as the membrane source for LC3 lipidation [5, 6]. ER‐phagy can alleviate endoplasmic reticulum stress and promote the transfer of STING proteins from the endoplasmic reticulum membrane to the autophagosome membrane through the cGAS/STING pathway [7]. Recent studies suggest that STING may be a promising therapeutic target in neurology [7, 8, 9]. STING triggers neuroinflammatory cascades that amplify nociceptive signaling. Inhibiting spinal STING–TBK1 signaling reduces ER stress and mechanical allodynia, positioning it as a potential target for neuropathic pain therapy. However, the different cell types and varying degrees of regulation of STING may lead to a paradoxical effect on pain and emotions in the neuropathic pain model. Moreover, the role of STING in regulating ER‐phagy in PrL neurons of SNL mice and its impact on pain and emotion‐related behavioral tests has not been explored.

In this study, our data indicated that ER‐phagy reduced excessive ER stress in the PrL of SNL mice. We found that the activation of STING and the cGAS/STING pathway is involved in ER‐phagy injury in the forelimb cortex, leading to neuropathic pain and emotional comorbidity. Regulating FAM134B and STING in PrL neurons altered pain and depression behaviors. Additionally, inhibiting the interaction between STING and LC3 regulates SNL‐induced pain and related emotional issues. This study suggests that the STING and cGAS/STING pathways could serve as therapeutic targets for neuropathic pain and associated depression.

## 2 Materials and Methods

### 2.1 Animals

The protocol of this study has been approved by the Animal Ethics Committee of China Medical University (Approval No. 2023PS015K). All surgeries were performed on mice under anesthesia with 4% chloral hydrate and sevoflurane, and all measures were taken to minimize animal pain. In this study, C57BL/6J mice (male, 6–8 weeks, 20–30 g, purchased from China Changsheng Biotechnology) were used to investigate neuropathic pain and related emotional disorders. According to the requirements of the feeding environment, these animals are individually housed in a controlled environment with a temperature of 23°C–25°C, humidity of 40%–70%, and a 12‐h light/dark cycle, with no other debris in the cage. Mice can have unrestricted access to food (C57BL/6J specific mouse food) and water and are randomly assigned to each experimental group. The experimenters were blind to the animal group.

### 2.2 Spinal Nerve Ligation (SNL)

According to the method initially proposed by Kim and Chung, after disinfection under anesthesia, an opening of about 2 cm is opened along the spine near the hip bone to separate the fascia and muscles, expose the L5 transverse process, carefully bite off the L5 transverse process with forceps, expose the L5 nerve, separate the left L5 spinal nerve with a glass needle, ligate it with 4–0 silk thread, and cut at the distal end of the ligation site. The surgical procedure of the sham surgery group was the same as that of the SNL group, but the spinal nerves were neither ligated nor transected.

### 2.3 Mechanical Paw Withdrawal Threshold (MWT)

Mechanical withdrawal threshold (MWT) testing was performed using von Frey filaments (Stoelting Company, Wood Dale, IL, USA). After adapting to the environment for 60 min, use a probe to vertically and uniformly stimulate the skin in the middle of the left hind foot of the rat. Record the pressure applied by the probe on the screen when the rat shrinks, which is the mechanical pain threshold. The stimulation duration is about 1 s, and the critical value for mice is 15 g. The characteristic of a positive reaction was that the hind paw retracts after stimulation, following the up‐and‐down testing procedure.

### 2.4 Thermal Paw Withdrawal Latency (TWL)

The thermal withdrawal latency (TWL) test is used to evaluate the sensitivity of paws to thermal stimuli. In a quiet environment, the room temperature remains constant at 20°C ± 2°C. After adapting to the environment for 60 min, the animals were placed in an organic glass room equipped with a fully automatic foot pain tester (BME‐410C, Shanghai, China). The left hind paw of mice was subjected to thermal stimulation. The experiment has a 5‐min interval and a duration of 20 s.

### 2.5 Forced Swimming Test (FST)

A mouse was placed in the FST water tank at a temperature of 25°C. The behavior was observed and recorded for 6 min, and the final 4 min of rest time were quantified using a tracking system (Noldus, China). The animal's center point was tracked to determine whether it was resting, and the threshold can be defined by itself. Additionally, activity detection can be used to observe changes from one video frame to the next. Subsequently, the mice were carefully removed from the water tank, dried with a towel, and had the water replaced in the tank after each treatment session.

### 2.6 Open Field Test (OFT)

Use OFT to assess anxiety and exercise levels. The arena consists of a 100 cm × 100 cm aluminum base and 45 cm high walls. The interior of the open field is painted white and equipped with detectors that use Noldus software to record and analyze animal behavior. Recorded parameters such as the distance traveled, the proportion of the central path, and the number of times the OFT passes through the central area, which accounts for half of the total area size. The experimental time was 10 min. After each experiment, clean the field with 75% ethanol.

### 2.7 Drug Delivery

Use a stereotactic instrument for PrL injection (AP: +1.94 mm, ML: −0.5 mm, DV: −2.5 mm). After anesthesia, the experimental animal was secured in a stereotactic device, with its head stabilized by ear rods and nose clips. Made a small incision on the scalp and used a stereotactic drill to drill holes at designated coordinates. Subsequently, the inducer or inhibitor was delivered through a glass needle via an injection pump. The applied compounds include rapamycin (RAP, autophagy inducer, CST, 0.2 μg/10 μL), 3‐MA (autophagy inhibitor, Selleck, 50 μM), 4‐PBA (ER stress inhibitor, Selleck, 200 μM), and tunicamycin (TM, ER stress inducer, Selleck, 25 μM). In the corresponding SNL + RAP, SNL + 3‐MA, SNL + 4‐PBA, and SNL + TM groups, 0.5 μL of each compound was injected on the day of surgery and postoperative days 4, 8, and 12. The sham surgery group underwent sham surgery and received a control PrL injection. The SNL + C group received SNL treatment and control injections. The sham+C group and SNL + C group received 0.5 μL of control (physiological saline) on the day of surgery and postoperative days 4, 8, and 12.

RU.521 (0.1 μ g/d, cGAS/STING inhibitor, catalog number inh‐RU‐521, InvivoGen, USA, 0.2 μ g/μ l) and 2′3‐ cGAMP (0.5 μ g/d, cGAS/STING ligand, catalog number tlrl‐nacga23, InvivoGen, USA, 1 μ g/μ l) were administered via Hamilton microsyringe on the day of surgery and postoperative days 4, 8, and 12 (subcutaneous injection on the back).

Ketamine (catalog number: 1709291) was purchased from Fujian Pharmaceutical Co. Ltd. in China. Inject intraperitoneally at a dose of 10 mg/kg 4 h before surgery and on postoperative days 4, 8, and 12. The dosage and concentration of the drug were determined based on preliminary experiments and published literature.

### 2.8 AAV Injection

The mice were anesthetized (4% chloral hydrate and sevoflurane) and secured in a stereotaxic apparatus (RWD Life Science, China). Body temperature was maintained at 36°C using a heating pad throughout the procedure. Once deep anesthesia was confirmed by the absence of a tail pinch response, the fur on the cranium was removed, and the skin was disinfected with 75% ethanol. A scalpel incision from 3 mm anterior to the bregma to 3 mm posterior to the lambda was made, and the skin was retracted via hemostats to expose the skull fully. The skull was cleared of tissue and roughened using cotton swabs to ensure clear visibility of the bregma and lambda. The stereotaxic ear bars and nose cone were adjusted to align the bregma and lambda on the same horizontal plane, as were two points 1.5 mm lateral to the midline on both sides of the skull.

We used stereotactic instruments for AAV virus injection. The injection needle was prepared by connecting it to a syringe containing the AAV virus solution (0.3 μL for PrL injection). AAV injections were performed on mice under anesthesia and slowly administered to the initial coordinates (AP: +1.94 mm, ML: −0.5 mm, DV: −2.5 mm) through a peristaltic pump. Then carefully pull the needle out of the brain after a 10‐min waiting period and suture the scalp incision with suture thread.

AAV2/9‐hSyn‐retreg1‐ZsGreen; AAV2/9‐hSyn‐retreg1‐RNAi (66270)‐ZsGreen; AAV2/9‐hSyn‐sting‐RNAi (116072–1)‐EGFP; AAV2/9‐hSyn‐sting (85068–1)‐EGFP; and AAV2/9‐hSyn‐sting∆atg8 (85082–1)‐EGFP were employed to modulate fam134b/retreg1 (Hanheng Technology, China) and sting (Jikai Technology, China) in this study. The AAV dosage was determined based on our preliminary experiments.

### 2.9 Western Blot

For Western blot analysis, the animals were anesthetized and euthanized using the quick blood loss method. Quickly removed the contralateral anterior lobe cortex (PrL) tissue and immediately froze it at −80°C. The samples were homogenized in ice‐cold RIPA lysis buffer (p0013B, Beyotime, China), incubated on ice with protease and phosphatase inhibitors (1:100, Solarbio, China) for 30 min, and then centrifuged at 14000 rpm for 40 min at 4°C. Then the supernatant was collected for further analysis. The protein concentration was measured using the BCA protein assay kit (Solarbio, China). The sample protein was separated using 12% SDS‐PAGE and transferred onto a PVDF membrane (GE, USA). Seal the membrane with 5% BSA (0.1% Tween‐20 in Tris‐buffered saline) at room temperature for 1 h. The primary antibody was incubated overnight at 4°C. After washing the primary antibody with TBST, the membrane was incubated with HRP‐conjugated secondary antibody at room temperature for 1 h. After washing with TBST, a chemiluminescence imaging system (GE, USA) c300, Azure Biosystems was used to capture the image.

The following antibodies were used: rabbit Grp78 (1:1000, Abcam, USA), rabbit PERK (1:1000), rabbit p‐PERK (1:500, CST, USA), rabbit ATF4 (1:500), rabbit ATF6 (1:100, Abcam, USA), rabbit IRE1 (1:100), rabbit p‐IRE1, rabbit cleaved caspase‐3 (1:1000, Abcam, USA), rabbit STING (available from CST, Abcam, and Thermo Fisher Scientific for 1:1000, CST, USA), rabbit p‐STING (1:1000), rabbit cGAS (1:1000, CST, USA), mouse GAPD H (1:8000, Solarbio, China) and goat anti rabbit/mouse IgG horseradish peroxidase (1:5000, Beyotime, China).

### 2.10 Immunoprecipitation

Samples were collected on postoperative day 7 (sham group and SNL group) and day 14 (control+SNL group, sting ∆ atg8 + SNL group). Perform immunoprecipitation testing according to the protocol provided in the Pierce IP/Co‐IP detection kit (catalog number 88828). The antibodies used include rabbit FAM134B antibody, rabbit STING, and mouse LC3. Evaluated protein expression through Western blot analysis.

### 2.11 Immunofluorescence (IF) Staining

The animals were deeply anesthetized and perfused with a 0.9% NaCl solution through the heart, followed by perfusion with 4% cold paraformaldehyde in 0.1 M PBS. Then extract the brain, fix it in 4% paraformaldehyde fixative for 24 h, and dehydrate it in 30% sucrose ddH2O at 4°C for at least 24 h. Subsequently, the brain was embedded, and the anterior cerebral cortex (PrL) was coronally sectioned to a thickness of 10 μm using a low‐temperature thermostat. To perform double immunostaining, coronal sections were also stained with anti‐NeuN (neuronal marker, 1:100, MAB377, Millipore, USA) and anti‐Grp78 (ER stress marker, 1:15, Abcam). Incubate with antibodies from the United States and then incubate with TRITC‐conjugated (1:200, Proteintech, China) and FITC‐conjugated (1:20, Proteintech, China) secondary antibodies. The cell nucleus was counterstained with DAPI (Beyotime, China) for 5 min.

### 2.12 Transmission Electron Microscopy (TEM) Staining

PrL tissues were carefully selected to minimize mechanical damage. Place a 1 mm^3^ tissue block into an EP tube containing TEM fixative for further fixation. Washed the tissue three times with PBS, each time for 15 min. After post‐fixation and dehydration at room temperature, the tissue was embedded in resin. Then, these resin blocks were cut into thin sections and stained for observation.

### 2.13 In Vivo Electrophysiological Recording

The following electrophysiological procedures were implemented to investigate the effects of the related AAVs on neural activity, as reflected by local field potentials (LFPs) in mice. Stereotactic implantation of a 30‐gauge stainless‐steel guide cannula targeted the PrL. The cannula was secured with dental cement, and the microwire electrodes were subsequently lowered to a 500 μm depth beneath the cannula aperture. Four skull screws were used for stabilization, with two screws also serving as the ground and reference electrodes. A custom‐designed head restraint device was affixed with dental cement. After a week of the postoperative recovery period, the mice were acclimated to the head fixation and recording conditions until they exhibited no signs of stress, such as teeth chattering, porphyrin staining, vocalization, and physical resistance to fixation, before the initiation of electrophysiological recordings.

Electrophysiological recordings were conducted within an enclosure to minimize ambient light and noise. The mice were positioned on a disk coated with an acoustic‐damping polymer (AMSZ16, Zongdiao, China) and mounted on a low–friction, silent rotor mechanism (2,585,858, JingKa, China). Continuous monitoring of eyelid movements and disk rotation ensured that recordings were obtained exclusively under conditions of alert wakefulness.

LFPs were recorded under both no stimulation and Von Frey filament stimulation conditions. Neural signals were filtered between 0.3 Hz and 7.5 kHz and amplified using a multi‐channel preamplifier (PBX Preamplifier; Plexon, Dallas, TX, USA). The amplified signals were then transferred to a digital multichannel acquisition processor (MAP; Plexon) and stored on the host computer's internal hard disk drive for offline analysis.

### 2.14 Statistical Analyzes

The results are presented in the form of mean ± standard error (SEM). Use IBM SPSS Statistics 22 software (SPSS Inc., New York, USA) or Prism 10.2 for analysis. All data were analyzed for normality and logarithmic normality using the Shapiro–Wilk test. Western blot, OFT, and FST data were analyzed using one‐way analysis of variance, followed by multiple comparison tests using Tukey's test (compared to each group) or Dunnett's test (compared to the control group) after a Shapiro–Wilk test. If the data do not exhibit a Gaussian distribution, the Kruskal–Wallis's test and Dunn's multiple comparison test are used to analyze the data. Use a non‐paired *t*‐test to analyze FST between the sham group and the SNL group. Use logarithmic (MWT/TWL) conversion to convert MWT and TWL data, confirm normality through the Shapiro–Wilk test, and analyze using two‐way ANOVA and Tukey multiple comparisons. A *p*‐value < 0.05 is considered statistically significant.

LFPs were extracted by subjecting broadband extracellular signals to a 300 Hz low–pass filtering process in both the anterograde and retrograde directions, employing a fourth–order Butterworth filter to minimize phase distortions. The LFPs were subsequently resampled to a standard sampling frequency of 1 kHz. Spectral analysis of LFPs was performed using a wavelet–based algorithm integrated into custom scripts that leverage the EEGLAB toolbox (https://sccn.ucsd.edu/eeglab/index.php). By employing the ‘time’ function from within the EEGLAB environment, an in–depth examination of the LFP spectral characteristics was facilitated. Mean trial power (MTP) analysis was implemented to evaluate the mean spectral power across trials, encompassing both stimulated and non‐stimulated conditions within individual recording sessions. The MTP values were extracted from the spectral–temporal domain and adjusted using a decibel (dB) baseline correction protocol standardized by EEGLAB. The gamma band power was determined by averaging the spectral power within the frequency range of 30–100 Hz.

## 3 Results

### 3.1 SNL Induced ER‐Phagy Impairment and Excessive ER Stress in the PrL of Mice

**FIGURE 1:**
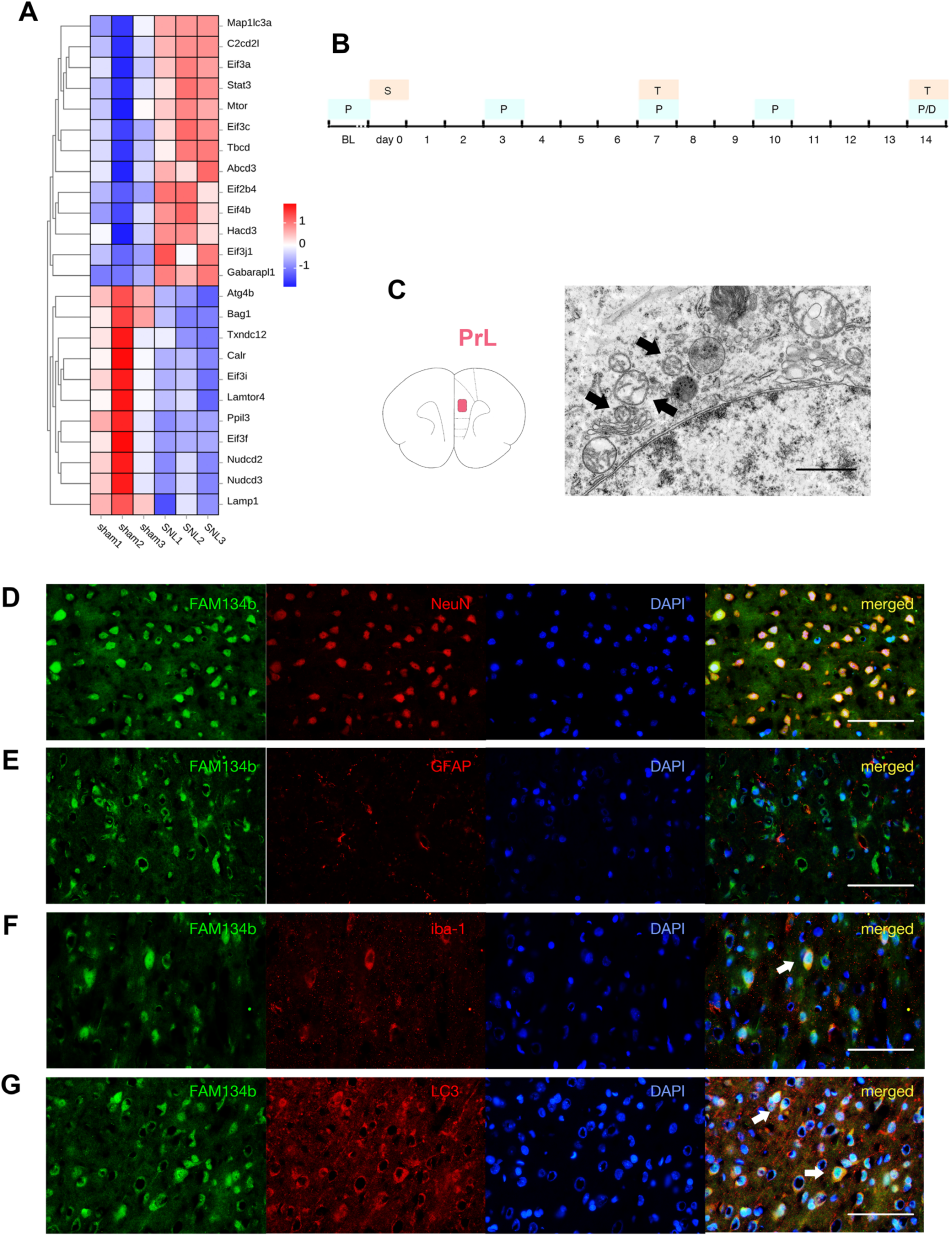
ER‐phagy was altered in the PrL of SNL mice. (A) Proteome sequencing results of sham and SNL groups. (B) Experimental design timeline; Abbreviation: S: surgery procedure; T: tissue collection; P: pain‐related behavior tests; D: depression‐related behavior tests (forced swimming test); I: PrL injection; BL: Baseline (2 days before surgery). (C) Electron microscopy showed representative samples taken from PrL of SNL (postoperative day 7) mice. Arrows indicated ER‐phagy. Scale bar: 10 μm, *n* = 3. (D‐G) Representative double immunofluorescence images of FAM134b and NeuN/GFAP/iba‐1/LC3 in PrL of SNL (SNL postoperative day 14) mice were presented. Scale bar: 100 μm, *n* = 3 per group.

Figure 1A showed some relevant proteomic results, where −1, 0, and + 1 represent log2 fold change (FC). The positive or negative values were used to determine whether the expression levels of these proteins have increased or decreased. The results indicated that changes in ER activity and autophagy levels were related to PrL in SNL mice. The experimental design and treatment schedule were depicted (Figure 1B). Electron microscopy of PrL in the SNL model showed that autophagosomes, indicated by arrows, degraded the endoplasmic reticulum (ER) (Figure 1C). These data suggested that ER‐phagy in PrL might be involved in the development of neuropathic pain. Immunofluorescence was used to identify a subpopulation of PrL cells expressing the ER‐phagy marker FAM134b. Figure 1D–1G showed the co‐localization of FAM134b with NeuN/Iba‐1/LC3, indicating that FAM134b was mainly expressed in the neurons and microglia of PrL in SNL mice.

**FIGURE 2:**
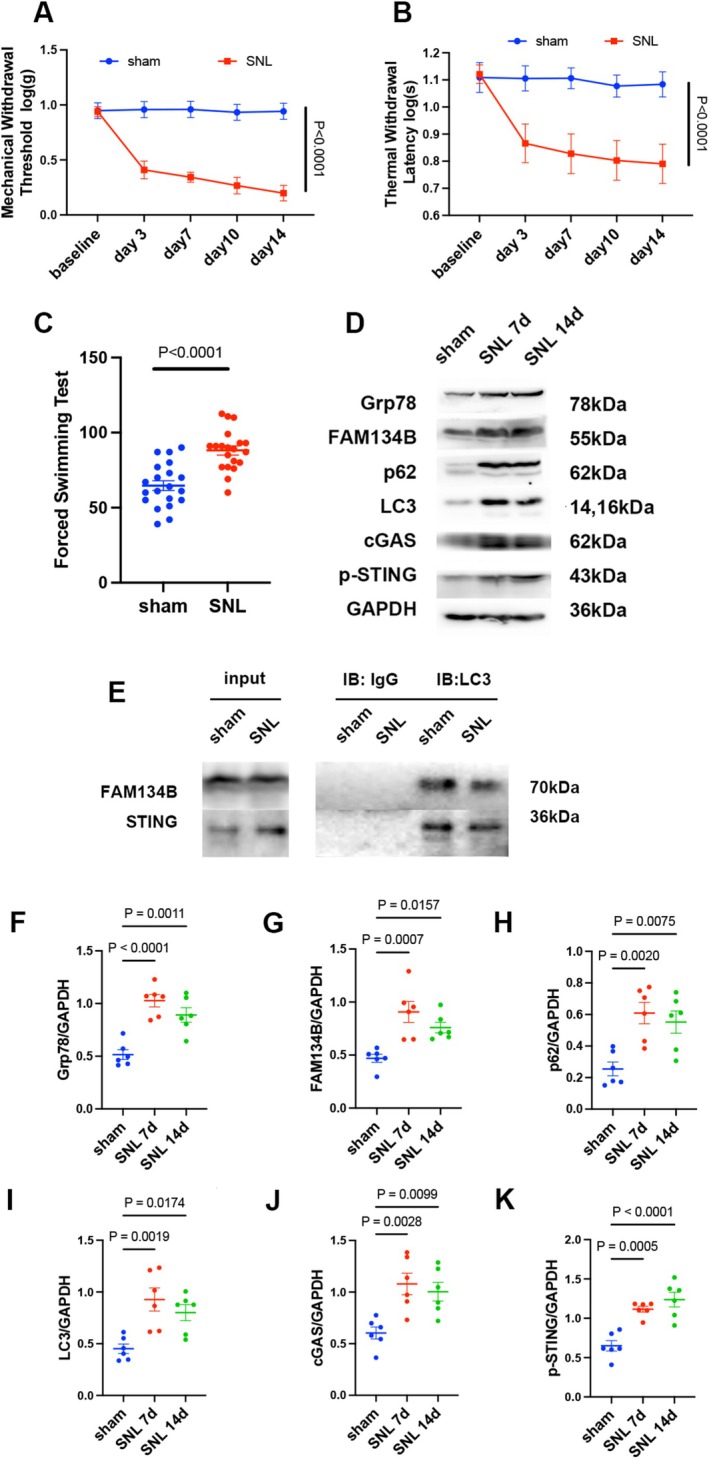
The cGAS/STING pathway and ER‐phagy levels were altered in the PrL of the SNL model. (A‐C) Comparisons of MWT, TWL, and FST in the sham and SNL groups. Two‐way ANOVA, MWT, *F*(4,120) = 224.3, *p* < 0.0001; TWL, *F*(4,120) = 60.26, *p* < 0.001, *n* = 16; unpaired *t* test with Welch's correction, FST, *t =* 5.310, df = 37.77, *p* < 0.0001, *n* = 20; (D) Western blot of PrL; (E) Immunoprecipitated LC3 samples interacted with STING and FAM134B antibodies in sham and SNL groups, *n* = 3; (F‐K) Quantifications of Western blot in PrL among the sham, (SNL postoperative day 7) and SNL14d group (SNL postoperative day 14) are shown, *n* = 6. The sham group received a sham operation of the SNL procedure; the SNL group received the SNL procedure. *p < 0.05, **p < 0.01, ***p < 0.001.

Previous studies have shown changes in spinal autophagy levels in the SNL model. We evaluated the role of ER‐phagy and ER stress in neuropathic pain in PrL and their comorbid behavioral manifestations related to pain‐induced emotions. Our research results indicated that spinal nerve ligation can lead to mechanical and thermal hypersensitivity reactions (Figure 2A,B). Through the time‐frequency analysis of EEG signals, we can observe that the brain electrical activity of the SNL model significantly increases compared with sham group (Figure S2A). Pain‐related behavior tests were conducted on the 2nd day before surgery (baseline) and on the 3rd, 7th, 10th, and 14th days after surgery. On the 14th day after surgery, SNL‐induced depressive behavior in mice was evaluated through the forced swimming test (Figure 2C). Representative Western blot of the relevant proteins was displayed (Figure 2D). Moreover, immunoprecipitation LC3 samples interacting with STING and FAM134B antibodies in the sham surgery group and SNL group showed that there was an LC3 bind to FAM134B and STING in the neuropathic pain model (Figure 2E). Compared with the sham surgery group, the expression levels of glucose‐regulated protein 78 (Grp78, an ER stress marker), FAM134b, LC3, and p62, which negatively correlated with autophagic activity, were significantly increased. In addition, the cGAS‐STING pathway was also activated considerably. (Figure 2F–2K). These results indicated an excessive increase in ER stress and impaired ER‐phagy in PrL neurons of SNL mice.

### 3.2 Endoplasmic Reticulum Stress and Autophagy Impairment Are Mutually Causal in the PrL of SNL Mice

To investigate the relationship between ER stress and ER‐phagy in PrL of SNL mice, we evaluated the expression of ER‐phagy proteins after ER stress inhibitors or inducers. After ER stress was suppressed, protein expression associated with this interaction was evaluated. Through TWL and MWT measurements, injection of 4‐phenylbutyric acid (4‐PBA, an ER stress inhibitor) into PrL produced significant analgesic effects (Figure 3.1A,B). It also alleviated depressive behavior in SNL mice (Figure 3.2C). Compared with untreated SNL counterparts, the expression of glucose‐regulated protein 78 (Grp78) and three unfolded protein response (UPR) pathways were downregulated after 4‐PBA administration, namely protein kinase RNA like ER kinase (PERK)/activated transcription factor 4 (ATF4), activated transcription factor 6 (ATF6), and inositol dependent enzyme‐1 α (IRE‐1)/c‐Jun N‐terminal kinase phosphorylation (p‐JNK) (Figure 3.1C). In addition, a decrease in p62 and an increase in LC3 and FAM134b were observed, indicating that ER stress inhibition promotes ER‐phagy in PrL of SNL mice. Compared with the SNL group, the cleavage of caspase‐3 was reduced, suggesting that 4‐PBA enhances ER‐phagy and promotes neuroprotection (Figure 3.1C). Protein Western blot quantification was displayed for the sham surgery group, sham+control group, SNL + control group, and SNL + 4‐PBA group (Figure 3.1D–N).

**FIGURE 3:**
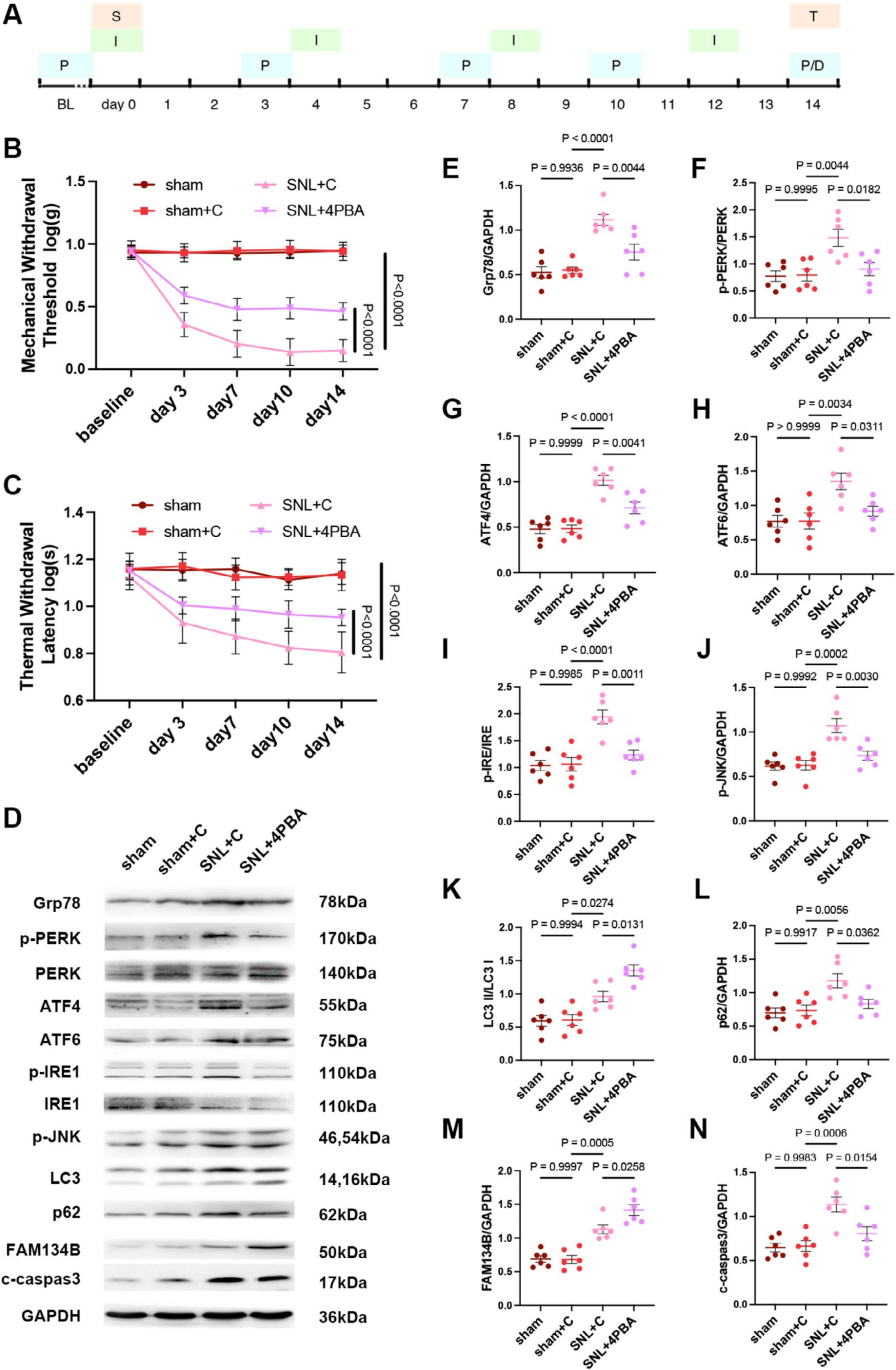
Effect of 4‐phenyl butyric acid (4‐PBA) and tunicamycin (TM) on pain‐related behavior tests and protein expression in the PrL of SNL model. (1A,1B) MWT and TWL tests. Two‐way ANOVA, MWT, *F* = 116.5; TWL, *F* = 20.56, *n* = 16. (1C) Representative Western blot of related proteins in sham, sham+C, SNL + C, and SNL + 4‐PBA groups are shown. Samples were harvested on postoperative day 14. (1D‐1 N) Quantifications of Western blot in sham, sham+C, SNL + C, and SNL + 4‐PBA groups are shown, *n* = 6. *p < 0.05, **p < 0.01, ***p < 0.001. (2A,2B) MWT and TWL in sham, sham+C, SNL + C and SNL + TM. Two‐way ANOVA, MWT, *F* = 90.71; TWL, *F* = 29.84, *n* = 16. (2C) FST data of SNL + V, SNL + PBA, SNL + TM, SNL + RAP, SNL + MA were demonstrated. *F =* 35.91, *n* = 18; (2D) displays the protein blotting of the sham surgery group, sham surgery+C group, SNL + C group, and SNL + TM group, with samples collected 14 days after surgery. (2E‐2O) Western blot in sham, sham+C, SNL + C, and SNL + TM groups were shown, *n* = 6, Samples were harvested at postoperative 14 days. *p < 0.05, **p < 0.01, ***p < 0.001.

In addition, compared with SNL mice, injecting tunicamycin (TM, an ER stress inducer) into PrL exacerbated mechanical and thermal hypersensitivity reactions (Figure 3.2A,B) and impaired the performance of the forced swimming test (Figure 3,2C). TM increased the levels of Grp78, p62, LC3, and cleaved caspase‐3 in PrL of SNL mice, while reducing the expression of FAM134b, indicating impaired ER‐phagy function. In addition, the expression levels of PERK/ATF4, ATF6, and IRE‐1/p‐JNK pathways were significantly increased, indicating that tunicamycin fully activated the three UPR pathways (Figure 3.2D–O).

To further explore the relationship between ER stress and autophagy, we modulated the autophagy level and observed the ER stress response. Through TWL and MWT measurements, injection of rapamycin, an autophagy inducer, into PrL resulted in significant analgesic effects. As mentioned earlier, the expression of PERK/ATF4, ATF6, and IRE‐1/p‐JNK was significantly downregulated. In addition, P62 decreased, and FAM134B increased, indicating that RAP promotes ER‐phagy and alleviates ER stress in PrL of SNL mice. In addition, the reduction of cleaved caspase‐3 (Figure S1A–S1C) indicated that RAP enhances ER‐phagy, thereby promoting neuroprotection. Compared with SNL mice, injecting 3MA into PrL exacerbated mechanical and thermal hypersensitivity reactions. 3MA increased the levels of Grp78, p62, LC3, and cleaved caspase‐3 in PrL of SNL mice, while reducing the expression of FAM134b, indicating impaired ER‐phagy function (Figure S1D–S1F). To sum up, autophagy alleviated overwhelming ER stress via the ER‐phagy process.

### 3.3 Regulating ER‐Phagy in PrL Neurons of SNL Mice Altered Pain‐Related and Emotion‐Related Behavioral Tests

**FIGURE 4:**
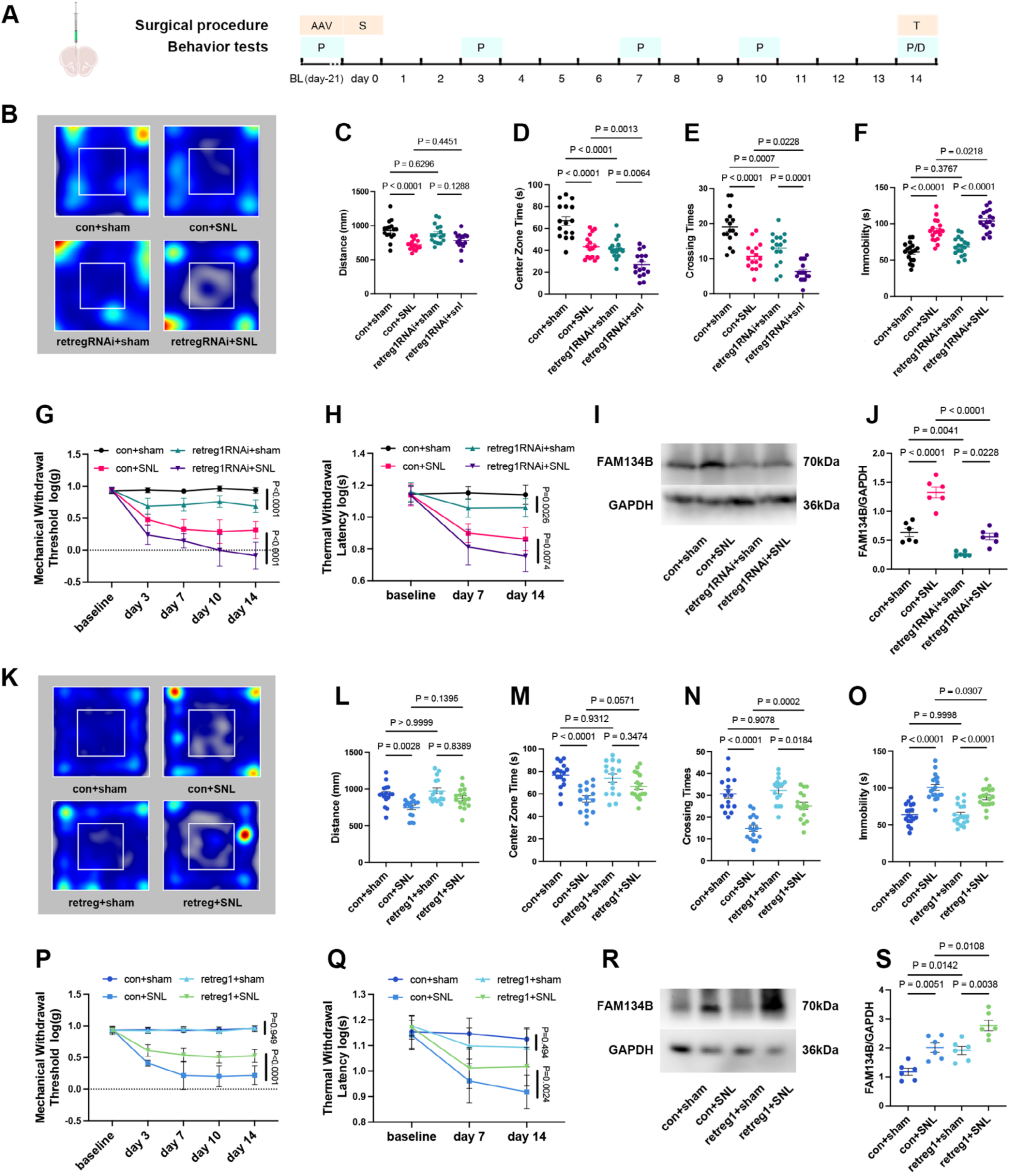
Effects of modulating PrL FAM134B expression via AAV‐retreg1RNAi and AAV‐retreg1 PrL injection on pain‐related and emotion‐related behavior tests and protein expression in the SNL model. (A) Experimental schedule for AAV injections. AAV: AAV injection; S: SNL procedure; P: pain behavior test; D: depression‐related tests. (B) Heat map of OFT. (C‐F) Quantification of OFT. ANOVA, distance: *F* = 7.599; center zone: *F* = 29.17; crossing times: *F* = 26.28. *n* = 16; (F): FST: ANOVA, *F* = 37.5, *n* = 18; (G) MWT: Two‐way ANOVA, *F* = 46.78, *n* = 16; (H) TWL: Two‐way ANOVA, *F* = 33.16, *n* = 16; (I, J) WB of PrL; (K) Heat map of OFT. (L‐N) Quantification of OFT, distance, Kruskal–Wallis's test: *P* = 0.0005; center zone, ANOVA: *F* = 9.464; crossing times, ANOVA, *F* = 22.74. *n* = 16; (O): FST: ANOVA, *F* = 28.98, *n* = 18; (P) MWT: Two‐way ANOVA, *F* = 55.21, *n* = 16; (Q) TWL: Two‐way ANOVA, *F* = 10.19, *n* = 16; (R, S) WB of PrL, *p* < 0.0001; *p < 0.05, **p < 0.01, ***p < 0.001.

Our data suggested that autophagy ameliorated ER stress via ER‐phagy. Next, we wonder whether ER‐phagy alleviates ER stress to attenuate neuropathic pain. We next assessed whether FAM134B, a key ER‐phagy protein, could modulate pain‐related behavioral tests. Our previous data suggested that ER‐phagy in PrL may serve as a feasible therapeutic target in SNL models through ER‐phagy inducers [10]. The immunofluorescence data in Figure 1 indicated that ER‐phagy mainly occurred in PrL neurons. We administered AAV2/9‐hSyn‐retreg1 and AAV2/9‐hSyn‐retreg1‐RNAi to modulate FAM134B expression to evaluate the analgesic and antidepressant effects of regulating FAM134B in PrL neurons. The experimental schedule for AAV injection is shown in Figure 4A. SNL or sham surgery was performed 3 weeks after AAV administration. Compared with the SNL group, the retreg1 group showed a decrease in brain electrical activity, and the retregRNAi group showed varying degrees of increased brain electrical activity compared to the sham surgery group (Figure S2B,S2C), suggesting altered FAM134B in neurons influenced the pain processing in the PrL. On the 14th day after SNL surgery, tests including MWT, TWL, FST, and OFT were conducted to evaluate hypersensitivity reactions, depression, and anxiety. Our results indicated that compared to the AAV‐con+SNL group, the AAV‐retreg1 + SNL group exhibited significant analgesic, anti‐anxiety, and antidepressant effects by enhancing FAM134B‐associated ER‐phagy (Figure 4). On the contrary, due to reduced ER‐phagy associated with FAM134B, pain‐related and emotional behavioral tests worsened in the AAV‐retreg1‐RNAi+SNL group.

### 3.4 STING In the PrL Neurons Participated in SNL‐Induced Pain and Emotion‐Related Behavior

Our data suggested ER‐phagy was essential in pain modulation, so we next assessed whether STING, an ER‐located protein that serves LC3 lipidation, modulated ER‐phagy in SNL mice.

To further investigate the effect of STING on pain and emotion‐related behavioral changes in neurons, we administered AAV2/9‐hSyn‐STING‐EGF to enhance STING expression and AAV2/9‐hSyn‐STING RNAi (116072–1)‐EGFP to inhibit it. Compared with the sham group, the sting group showed varying degrees of increased brain electrical activity (Figure S2D). This further confirms the above viewpoint. Our research findings indicated that the elevated expression of STING in PrL neurons exacerbates the comorbidity of pain and emotion (Figure 5.1A–J). Considering the direct interaction between STING and LC3 as shown in Figure 2E, we investigated whether blocking this interaction through AAV2/9‐hSyn‐STING ∆ Atg8 EGFP would affect the behavior in the SNL model. Compared with the sham+vehicle group, the sting△atg8 group showed varying degrees of increased brain electrical activity (Figure S2E). OFT, FST, MWT, and TWL suggested that the sting△atg8 deteriorated pain‐related behavior. WB confirmed the AAV transfection efficiency. The results indicated that disrupting STING and LC3 binding worsened neuropathic pain and emotional comorbidity (Figure 6.2A–K). Previous studies by Ji et al. have shown that *Sting*KO mice exhibit a higher pain threshold, and STING activation of microglia can alleviate neuropathic pain in mice after nerve injury [11]. We explored the effect of reduced STING expression in PrL neurons of SNL mice using AAV2/9‐hSyn‐sting RNAi‐EGFP. Compared with the SNL group, the stingRNAi group showed a decrease in brain electrical activity (Figure S2F). Our data indicated that the decrease in STING expression in PrL neurons of SNL mice improves MWT, TWL, FST, and OFT outcomes (Figure 5.3A–J), suggesting that STING downregulation produces analgesic, anti‐anxiety, and antidepressant effects in SNL mice.

**FIGURE 5:**
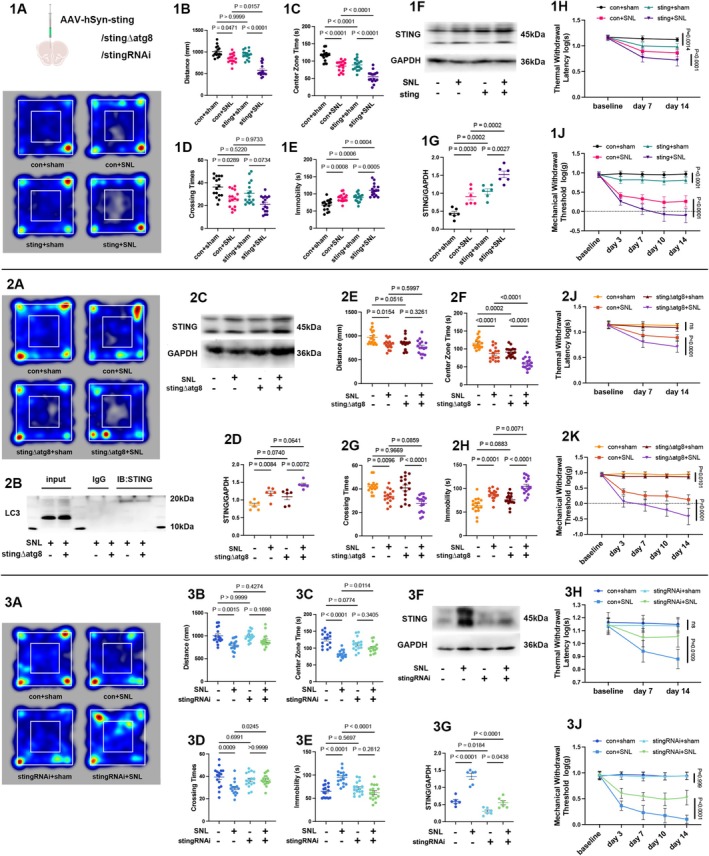
Effect of RU521 and 2′3‐cGAMP on pain‐related behavior tests and protein expression in the PrL of SNL model. (A) Experimental design and treatment schedule; Abbreviation: S: surgery procedure; T: tissue collection; P: pain‐related behavior tests; D: depression‐related behavior tests; R: RU.521 injection; K: ketamine administration; G: 2′3‐cGAMP administration; BL: Baseline (2 days before surgery). (B, C) Two‐way ANOVA, MWT, *F* = 67.90, *n* = 16; TWL, *F* = 18.26, *n =* 16; (D) Kruskal–Wallis test for FST, *p* = 0.0005, *n* = 18. (E‐K) WB results. Sham+V: Sham SNL procedure+ ketamine vehicle+ cGAMP vehicle; SNL + V: SNL+ ketamine vehicle+ cGAMP vehicle; SNL + K + G: SNL + ketamine+ cGAMP. (L) Two‐way ANOVA, MWT, *F* = 37.79, *n* = 16; (M) TWL, *F* = 21.96, *n* = 16. (N) One‐way ANOVA, FST, *F* = 19.61, *n* = 18. (O‐T) WB results. *p < 0.05, **p < 0.01, ***p < 0.001.

**FIGURE 6:**
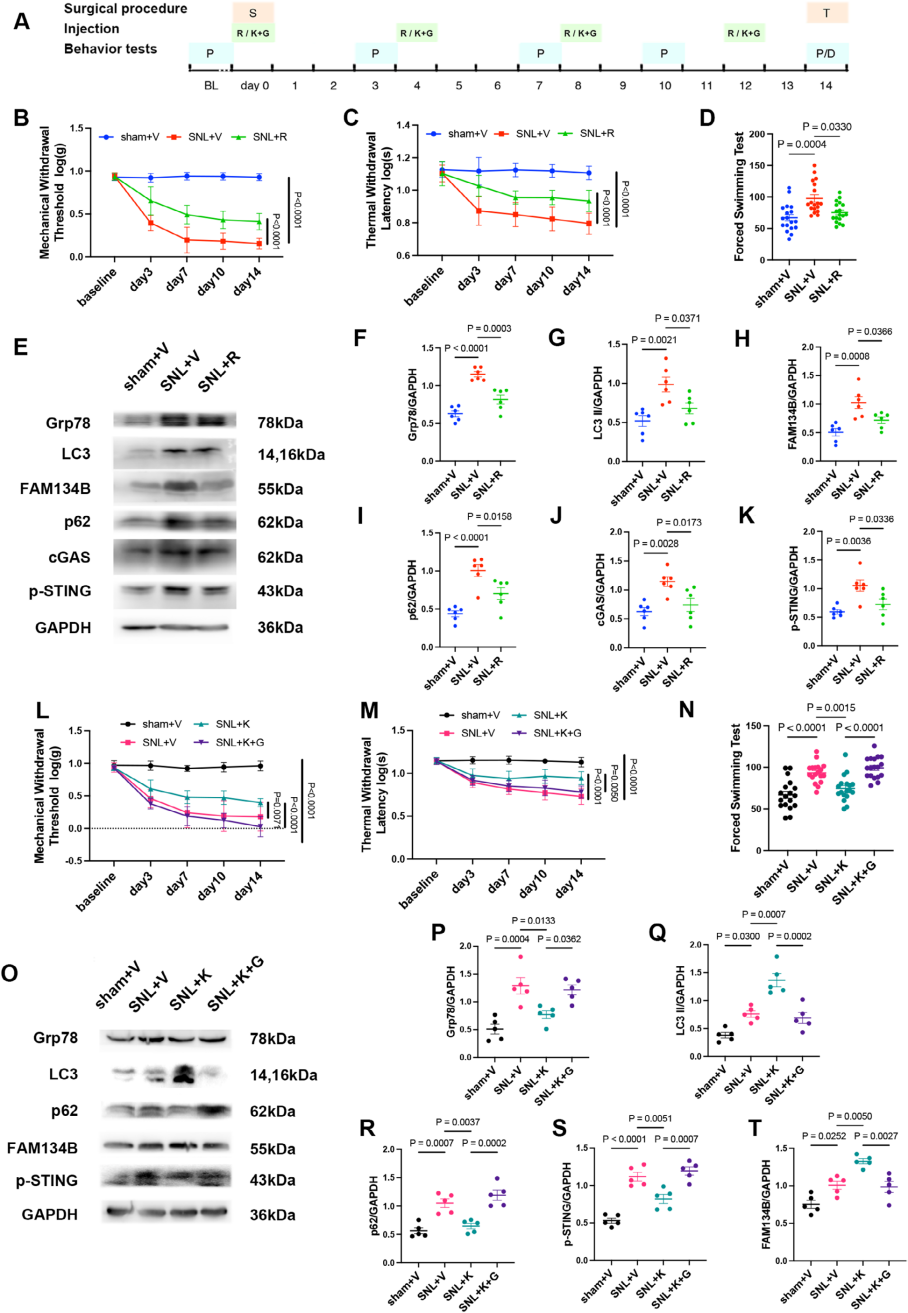
Effects of modulating PrL STING expression via AAV‐sting, AAV‐sting∆atg8, and AAV‐stingRNAi PrL injection on pain‐related and emotion‐related behavior tests and protein expression in the SNL model. (1A) AAV injection diagram and Heat map of OFT; (1B‐1D) Quantification of OFT, distance, Krusakal‐Wallis, *p* < 0.0001; center zone: ANOVA, *F* = 42.09; crossing time: Krusakal‐Wallis, *p* = 0.0003. *n* = 16; (1E) FST: ANOVA, *F* = 23.20, *n* = 16; (1F‐1G) WB results; (1H) TWL: ANOVA, *F* = 39.72, *n* = 16; (1 J) MWT: Two‐way ANOVA, *F* = 70.62, *n* = 16; (2A) Heat map of OFT; (2B) Co‐IP (2C‐2D) WB results, WB ANOVA, *n* = 5, *F* = 10.79, *p* = 0.0004. (2E‐2G) Quantification of OFT, ANOVA, distance: *F* = 6.676, *p* = 0.0062; center zone: ANOVA, *F* = 34.68, *p* < 0.0001; crossing time, *F* = 13.98. *n* = 16; (2H) FST: ANOVA, *F* = 23.26, *n* = 16; (2 J) TWL: ANOVA, *F* = 27.20, *n* = 16; (2 K) MWT: Two‐way ANOVA, *F* = 78.01, *n* = 16; (3A) Heat map of OFT; (3B‐3D) Quantification of OFT, distance: Kruskal–Wallis, *p* = 0.0001; center zone: ANOVA, F = 18.13; crossing time: ANOVA, F = 5.991. *n* = 16; (3E) FST: ANOVA, F = 15.97, *n* = 16; (3F‐3G) WB results; (3H) TWL: ANOVA, F = 15.03, *n* = 16; (3 J) MWT: Two‐way ANOVA, F = 64.95, *n* = 16; *p < 0.05, **p < 0.01, ***p < 0.001. Figure S1 Effect of rapamycin (RAP) and 3‐methyladenine (3‐MA) on pain‐related behavior tests and protein expression in the PrL of SNL model. (1A,1B) MWT and TWL of sham surgery, sham +C, SNL + C, and SNL + RAP. *n* = 12. (1C) WB of the sham surgery group, sham +C group, SNL + C group, and SNL + RAP group, with samples collected 14 days after surgery. *n* = 5. (1D,1E) MWT and TWL of sham surgery, sham+C, SNL + C, and SNL + 3MA. *n* = 12. (1F) WB of the sham surgery group, sham+C group, SNL + C group, and SNL + 3MA group, with samples collected 14 days after surgery. *n* = 5. Figure S2 (A‐F) Local field potential under no stimulation and von Frey stimulation. Time‐frequency analysis chart of electroencephalogram signals. (1A) The sham group and the SNL group. (1B) SNL + V and SNL + stingRNAi. (1C) sham+V and sham +sting. (1D) sham+V and sham +sting △ atg8. (1E) sham+V and sham+retrogRNAi. (1F) SNL group+V and SNL + retreg. *n* = 5.

### 3.5 cGAS/STING Pathway Regulated ER‐Phagy to Modulate SNL‐Induced Pain‐ and Emotion‐Related Behaviors

To investigate the effect of the STING pathway on ER‐phagy levels in SNL mice, cGAS/STING inhibitor, RU521, was administered in the PrL of SNL mice. Compared with the SNL + vehicle group, the RU521 treatment group showed significant analgesic effects, as demonstrated by MWT and TWL experiments (Figure 6A–6C). In addition, compared with the SNL + vehicle group, RU521 alleviated depression‐related behaviors (Figure 6D). Compared with the SNL + vehicle group, RU521 treatment resulted in a significant decrease in cGAS and phosphorylated STING levels. Compared with the SNL + vehicle group, the Grp78, LC3, FAM134b, and p62 of the SNL + RU521 group decreased, suggesting ER‐phagy impairment was attenuated after STING pathway inhibition (Figure 6E–6K).

Our previous data suggested that ketamine might affect SNL via STING. We wonder whether the STING pathway inducer can worsen SNL‐induced pain‐related behavior and ameliorate the effects of ketamine treatment. Our research findings indicate that ketamine improved the results of MWT, TWL, and FST, while increasing ER‐phagy (Figure 6L–6N). Compared with SNL mice, ketamine treatment resulted in a decrease in the expression levels of Grp78, p62, and phosphorylated STING (p‐STING), and an increase in the levels of LC3 and FAM134B (Figure 6O). In contrast, the cGAS/STING pathway agonist 2′3‐cGAMP alleviated the analgesic and antidepressant effects of ketamine (Figure 6L–6N). In the PrL of SNL mice treated with 2′3‐cGAMP, the expression levels of Grp78, p62, and p‐STING significantly increased, while LC3 and FAM134B significantly decreased (Figure 6O–6T). These findings suggested that the analgesic and antidepressant properties of ketamine were mediated through the cGAS/STING pathway, thereby affecting the ER‐phagy levels in SNL mice.

## 4 Discussions

Autophagy is crucial in the brain's response to stress in central nervous system diseases [12]. Our previous research has shown that endoplasmic reticulum stress leads to dysfunction of ER‐phagy in the spinal cord of SNL rats, affecting the nociceptive decline system [10]. Studies have emphasized that nerve damage or harmful stimuli can trigger long‐term presynaptic and postsynaptic plasticity of PrL, leading to negative emotions associated with neuropathic pain [2, 3, 4, 5, 6, 7, 8, 9, 10, 11, 12, 13].

In this study, we investigated the co‐regulation of ER stress and ER‐phagy in the PrL of SNL mice. We also measured the levels of cleaved caspase 3 to evaluate apoptosis in the PrL of SNL mice. Our research results indicated that an increase in ER‐phagy and a decrease in ER stress contributed to the pro‐survival effect of SNL mice. In contrast, inhibiting ER‐phagy enhances ER stress, leading to an increase in cleaved caspase‐3 levels, indicating a pro‐apoptotic effect. These results indicated that enhanced ER‐phagy was a protective advantage against neuropathic pain.

FAM134B (RETREG1) contains the LC3 interaction region (LIR) domain, which mediates autophagic cargo through ER‐phagy [14, 15, 16]. Research has shown that FAM134B is crucial in neurological disorders and inflammatory responses [12, 13, 14, 15, 16, 17, 18, 19]. The loss‐of‐function mutation in FAM134B (RETREG1) leads to HSAN2B (Hereditary Sensory and Autonomic Neuropathy Type IIB), a rare genetic disease characterized by a gradual decrease in pain, temperature, and tactile sensation [12, 13, 14, 15, 16]. Knockout of FAM134B leads to changes and apoptosis of the cis Golgi apparatus in dorsal root ganglion neurons, indicating its potential role in nociceptive regulation [12, 13, 14, 15, 16, 17, 18, 19, 20, 21]. After injection of RAP or 4‐PBA, FAM134B expression was upregulated, indicating that enhanced ER‐phagy was involved in the pain process. Our immunofluorescence data indicated that FAM134B was mainly co‐localized with NeuN in SNL mice. We further investigated whether FAM134B in neurons regulated neuropathic pain and related emotional complications by injecting AAV tools through stereotactic devices. Our research findings indicated that the reduced expression of FAM134B in PrL neurons exacerbated neuropathic pain, anxiety, and depression. On the contrary, enhancing the expression of FAM134B in PrL neurons of SNL mice provided anti‐anxiety, anti‐depression, and analgesic effects, indicating that ER‐phagy in PrL was crucial for regulating neuropathic pain and emotional comorbidity.

Our co‐immunoprecipitation data indicated that LC3 was associated with FAM134B and STING in the PrL of SNL mice. Therefore, we investigated whether STING is involved in regulating SNL‐induced PrL ER‐phagy alterations. Research has shown that STING is associated with diseases, including pain processes. The functional acquisition mutation in the STING encoding gene TMEM173 leads to SAVI (interferon gene stimulatory factor associated with vascular disease in infancy) [22, 23]. It is known that Janus kinase (JAK) inhibitors can alleviate symptoms of SAVI and have also shown analgesic effects in neuropathic pain models [24, 25]. In addition, the activation of STING is associated with chronic neurodegenerative diseases and the regulation of microglial cell activity [25]. Enhancing the STING pathway in microglia can activate TREM2 (a transmembrane receptor), thereby alleviating neuroinflammation in the later stages of Alzheimer's disease models [26, 27]. It is worth noting that activation and inhibition of the STING may play different roles in cancer treatment [28, 29, 30]. Recently, there has been increasing interest in the potential of the STING pathway as a therapeutic target, despite the conflicting roles of STING in relation to immune responses in the same or different diseases [5, 6, 7, 8, 9, 10, 11, 12, 13, 14, 15, 16, 17, 18, 19, 20, 21, 22, 23, 24, 25, 26, 27, 28, 29, 30, 31, 32]. A study has shown that STING is upregulated in CCI and SNI models, and the activation of STING in microglia can alleviate neuropathic pain [11]. This has prompted research on whether STING in PrL neurons affects pain and emotion‐related behaviors. We used AAV tools to manipulate the expression of STING in PrL neurons. The results indicated that the increased expression of STING in PrL neurons exacerbated pain and emotional behavior. On the contrary, reducing the expression of STING in PrL neurons exacerbated pain and emotion‐related behaviors. Our data suggested that STING regulated ER‐phagy by interacting with LC3, thereby altering pain and mood. After injecting AAV into PrL to sting ∆ atg8, evaluations such as MWT, TWL, FST, and OFT showed that the situation worsened, indicating that excessive STING required autophagy to restore the homeostasis of PrL neurons in the SNL model. Research has shown that *Sting*KO mice exhibited higher pain thresholds, and activating small glial cells STING reduced neuropathic pain in the mouse spinal cord [5, 6, 7, 8, 9, 10, 11, 12, 13, 14, 15, 16, 17, 18, 19, 20, 21, 22, 23, 24, 25, 26, 27, 28, 29, 30, 31, 32, 33]. Our data suggested that downregulating STING in PrL neurons of SNL mice can reduce neuropathic pain and emotional comorbidity. It is worth noting that different cell types may exhibit different responses to interventions. For example, GABAergic neurons may be particularly sensitive to drug therapy for neuropathic pain.

The cGAS/STING signaling pathway is crucial for activating anti‐infective pro‐survival mechanisms. However, excessive or long‐term activation of this pathway can lead to tissue damage and immunopathology. The activation of STING induces negative feedback through p‐TBK1 and p62‐dependent mechanisms [5, 6, 7, 8, 9, 10, 11, 12, 13, 14, 15, 16, 17, 18, 19, 20, 21, 22, 23, 24, 25, 26, 27, 28, 29, 30, 31, 32, 33, 34, 35]. Although the cGAS/STING pathway has become a potential therapeutic target, its role in neuropathic pain is still unclear. To evaluate whether the cGAS/STING pathway affects ER stress‐induced ER‐phagy in PrL of SNL mice, RU521 was used to inhibit the cGAS/STING pathway, while 2′3‐cGAMP was administered to activate the cGAS/STING pathway. Our results indicated that inhibiting the cGAS/STING pathway can not only alleviate neuropathic pain but also exert antidepressant effects. The significant decrease in cGAS and p‐STING indicated that RU521 enhanced ER‐phagy levels by downregulating the cGAS/STING pathway. It is worth noting that compared with the SNL + V group, the Grp78, LC3, p62, cGAS, and p‐STING in the SNL + *R* group were significantly reduced, while FAM134b was significantly increased. The increase of FAM134b was associated with the decrease of LC3 and p62 levels, indicating enhanced degradation of autophagosomes captured by the ER. The data also showed that RU‐521 improved SNL‐induced hypersensitivity reactions and depression. Previous data suggested that upregulating the cGAS/STING pathway through intrathecal injection of 2′3‐cGAMP can counteract the therapeutic effects of dexmedetomidine and ketamine [10]. In this study, we further revealed that the sustained and excessive activation of the cGAS/STING pathway in PrL of SNL mice counteracted the benefits of ketamine treatment, which significantly alleviated neuropathic pain and emotional comorbidities. Compared with the SNL + K group, Grp78, p62, and p‐STING were reduced in PrL, while LC3 and FAM134b were reduced. These results indicated that activation of the cGAS/STING pathway in SNL mice negated the analgesic and antidepressant properties of ketamine, leading to increased ER stress and impaired ER‐phagy. Overall, our data confirmed that the cGAS/STING pathway played a critical role in SNL‐induced neuropathic pain, and the regulation of STING provided analgesic and antidepressant advantages.

This study suggested that ER‐phagy alleviated neuropathic pain by reducing ER stress, thereby providing neuroprotection. In addition, manipulating the STING pathway and ER‐phagy in PrL neurons through FAM134B significantly influenced pain and emotion‐related behaviors. This study evaluated STING in PrL neurons of SNL mice, emphasizing its potential as a therapeutic target for neuropathic pain. However, this study has limitations. As mentioned earlier, altering the regulation of STING through different molecular mechanisms, cell types, or pain circuits may lead to different behavioral outcomes. It should be discussed whether different genders interact with specific neural circuits related to the STING pathway in further research. Our team plans to identify specific cell types or circuits affected by STING in vivo and elucidate the relevant mechanisms in future research. Subsequent studies should examine how the pharmacological or interventional treatment of neuropathic pain affects STING and ER‐phagy in neural circuits.

## Funding

This work was supported by the National Natural Science Foundation of China, 82301407. Applied and Basic Research Program of Liaoning Province (Joint Program), 2022JH2/101500058. Nature Science Foundation of Liaoning Province, 2023‐MS‐178. Outstanding Scientific Fund of Shengjing Hospital, 202208.

## Ethics Statement

The protocol of this study has been approved by the Animal Ethics Committee of China Medical University (Approval No. 2023PS015K).

## Conflicts of Interest

The authors declare no conflicts of interest.

## Supporting information


**Figure S1:** Effect of rapamycin (RAP) on pain‐related behavior tests and protein expression in the PrL of SNL model. (A, B) MWT and TWL in sham, sham+C, SNL + C and SNL+ RAP. *n* = 12 per group. (C) Representative Western blot of related proteins in sham, sham+C, SNL + C, and SNL+ RAP groups are shown. Samples were harvested on postoperative day 14, *n* = 6. *p < 0.05, **p < 0.01, ***p < 0.001. Effect of 3‐methyladenine (3‐MA) on pain‐related behavior tests and protein expression in the PrL of SNL model. (D, E) MWT and TWL in sham, sham+C, SNL + C and SNL + 3‐MA. *p < 0.05, **p < 0.01, ***p < 0.001. *n* = 12. (F) Representative Western blot of related proteins in sham, sham+C, SNL + C, and SNL + 3‐MA groups are shown, *n* = 6.

## Data Availability

The data that support the findings of this study are available from the corresponding author upon reasonable request.
