# Supplementary material for: STING Modulating ER‐Phagy in the Prelimbic Cortex Neurons Contributed to Neuropathic Pain and Emotional Comorbidity
Source: CNS Neurosci Ther. 2026 Jun 22;32(6):e70995. doi: 10.1002/cns.70995 (PMC13287086; doi:10.1002/cns.70995)
Supplement: Supplementary file 1 — Figure S1: Effect of rapamycin (RAP) on pain‐related behavior tests and protein expression in the PrL of SNL model. (A, B) MWT and TWL in sham, sham+C, SNL + C and SNL+ RAP. n = 12 per group. (C) Representative Western blot of related proteins in sham, sham+C, SNL + C, and SNL+ RAP groups are shown. Samples were harvested on postoperative day 14, n = 6. *p < 0.05, **p < 0.01, ***p < 0.001. Effect of 3‐methyladenine (3‐MA) on pain‐related behavior tests and protein expression in the PrL of SNL model. (D, E) MWT and TWL in sham, sham+C, SNL + C and SNL + 3‐MA. *p < 0.05, **p < 0.01, ***p < 0.001. n = 12. (F) Representative Western blot of related proteins in sham, sham+C, SNL + C, and SNL + 3‐MA groups are shown, n = 6. [file CNS-32-e70995-s001.docx]

Supplementary material

**Effect of rapamycin and** [**3-methyladenine**](http://www.baidu.com/link?url=GMq8BvS9lJRCdooULVBBWHvoGCtCnsIOkwT4gYF1hG4IsteSNkbY1tdHfMHyorloSLAYg2tHcRuW2OtD92-4xK) **injections on nociceptive behavior in SNL model**

To investigate whether ER-phagy provides a feedback mechanism to ER stress, rapamycin (RAP, autophagy inducer) was administrated intrathecally. Our data demonstrated that rapamycin led to an analgesic effect measured with MWT and TWL. Rapamycin administration led to Grp78, p62, cleaved caspase3 and PERK/ATF4, ATF6, IRE-1/p-JNK pathways down-regulation. The expressions of LC3 and FAM134b increased. RAP ameliorated ER stress and UPR via enhancing ER-phagy in PrL. Significant further aggravation of pain was observed measured with MWT and TWL in [3-methyladenine](http://www.baidu.com/link?url=GMq8BvS9lJRCdooULVBBWHvoGCtCnsIOkwT4gYF1hG4IsteSNkbY1tdHfMHyorloSLAYg2tHcRuW2OtD92-4xK) (3-MA, autophagy inhibitor) treating mice. The expressions of Grp78, LC3, p62, and cleaved caspase-3 were increased. Also, PERK/ATF4, ATF6, and IRE-1/p-JNK pathways were activated significantly. Besides, 3-MA intrathecal administration led to FAM134b down-regulation.


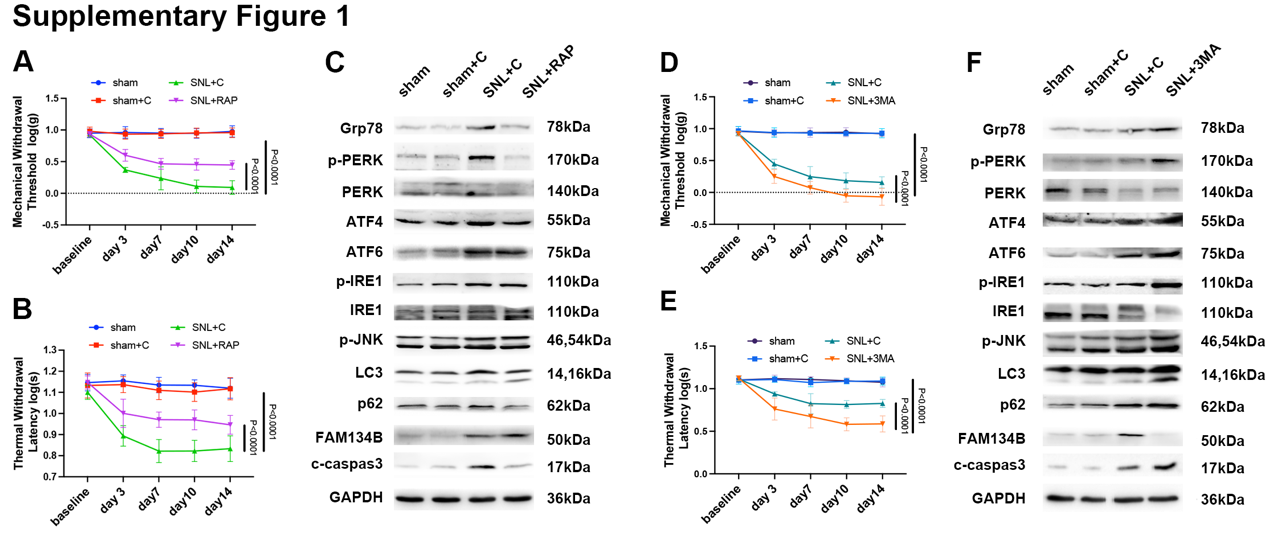


Supplementary Figure 1 legend

Effect of rapamycin (RAP) on pain-related behavior tests and protein expression in the PrL of SNL model. (A, B) MWT and TWL in sham, sham+C, SNL+C and SNL+ RAP. *n*=12 per group. (C) Representative Western blot of related proteins in sham, sham+C, SNL+C, and SNL+ RAP groups are shown. Samples were harvested on postoperative day 14, *n*=6. **P*<0.05, ***P*<0.01, ****P*<0.001. Effect of [3-methyladenine](http://www.baidu.com/link?url=pXiflwJSntGHyPwHrjFJOf1Lkc-PH7Wbscna7h9l3WdsdnSOKD9DMvFcPMwbtTClClLbCVz7KQzBr6e9EdNAkK) (3-MA) on pain-related behavior tests and protein expression in the PrL of SNL model. (D, E) MWT and TWL in sham, sham+C, SNL+C and SNL+3-MA. **P*<0.05, ***P*<0.01, ****P*<0.001. *n*=12. (F) Representative Western blot of related proteins in sham, sham+C, SNL+C, and SNL+3-MA groups are shown, *n*=6.
